# Dataset of de novo transcriptome assembly of Rhizome in *Curcuma aeruginosa* Roxb

**DOI:** 10.1016/j.dib.2023.109254

**Published:** 2023-05-23

**Authors:** Devit Purwoko, Imam Civi Cartealy, Siti Zulaeha, Teuku Tajuddin, Gemilang Rahmadara, Nurul Fitri Hanifah, Endah Dwi Hartuti, Hayat Khairiyah, Amila Pramisandi, Yusuf Sigit Ahmad Fauzan, Suparjo, Rikania Reninta, Anna Safarrida, Sobir

**Affiliations:** a Research Center for Genetic Engineering, Research Organization for Life Sciences and Environment, National Research and Innovation Agency. Sukarno's science and technology area Cibinong Bogor West Java 16911, Indonesia; b Research Center for Computation, Research Organization for Electronics and Informatics, National Research and Innovation Agency. Sukarno's science and technology area Cibinong Bogor West Java 16911, Indonesia; c Research Center for Horticulture and Plantation, Research Organization for Life Sciences and Environment, National Research and Innovation Agency. Sukarno's science and technology area Cibinong Bogor West Java 16911, Indonesia; d Research Center for Applied Microbiology, Research Organization for Life Sciences and Environment, National Research and Innovation Agency. Sukarno's science and technology area Cibinong Bogor West Java 16911, Indonesia; e Research Center for Plant Conservation, Botanical Gardens and Forestry, Research Organization for Life Sciences and Environment, National Research and Innovation Agency. Sukarno's science and technology area Cibinong Bogor West Java 16911, Indonesia; f Research Center for Medicinal Raw Materials and Traditional Medicines, Research Organization for Life Sciences and Environment, National Research and Innovation Agency. Sukarno's science and technology area Cibinong Bogor West Java 16911, Indonesia; g Department of Agronomy and Horticulture, Faculty of Agriculture, IPB University, Bogor, Indonesia

**Keywords:** *Curcuma aeruginosa*, Rhizome, Medicinal plant, Saponin, Transcriptomics

## Abstract

*Curcuma aeruginosa* Roxb. is an Indonesian traditional medicinal plant of the Zingiberaceae family. *C. aeruginosa* is known to have anticancer activity, especially in the rhizomes. Despite many studies on the phytochemical content of this plant with antioxidant and anticancer activity, transcriptomic studies are still limited in terms of genetic information. We ran transcriptome of *Curcuma aeruginosa* using a paired-end Illumina NextSeq 550 with PE150 mode and generating 12.8 GB of raw data. Raw reads have been filed with NCBI under project number PRJNA918644. This dataset allowed us to identify genes associated with biosynthetic pathways of anticancer drugs. Transcriptome data can also be used to develop new EST-SSR and SNP markers for use in plant breeding programs.

**Specifications Table**

**Table:** 

Subject	Agricultural and Biological Sciences
Specific subject area	Medicinal Plant Transcriptomics
Type of data	Table, text file
How the data were acquired	Illumina NextSeq 550 PE150 platform.
Data format	Raw Sequencing Reads, Table
Description of data collection	The rhizome of *C. aeruginosa* from Kebumen accession was obtained from 6 months old plant. Three types of rhizomes (primary, secondary and tertiary) were used in RNA extraction. RNA of rhizome samples was extracted using GeneAll® Ribospin™ Plant (GeneAll Biotechnology Co., Ltd.) and bulked up for RNA sequencing.
Data source location	Rhizome samples were collected at: • Institution: Laboratory for Biotechnology • City/Town/Region: Building 630, Puspiptek Serpong South Tangerang, Banten • Country: Indonesia
Data accessibility	Repository name: NCBI Sequence Read Archive (SRA) Data identification number: PRJNA918644 Direct URL to data: https://www.ncbi.nlm.nih.gov/sra/PRJNA918644 Repository name: Mendeley Data Direct URL to data: https://data.mendeley.com/datasets/79gzvszgjm/1

## Value of the Data

• These data provide transcriptome of *Curcuma aeruginosa* Roxb. from the rhizome.
• These data are useful for obtaining molecular markers such as microsatellites and single nucleotide polymorphisms for breeding and selection of new cultivars of *Curcuma aeruginosa* Roxb. and related genera.
• These data are also useful for studying differentially expressed genes (DEGs) and various signaling pathways. This will play an important role in discovering putative genes that use therapeutics in that species and related genera.
• These data can also be applied to identify different metabolic pathways in *Curcuma aeruginosa* Roxb. From the rhizome, as it is also an important medicinal plant.

## 1 Objective

*C. aeruginosa* has a rhizome that resembles turmeric and has a dark or blackish color [1]. *Curcuma aeruginosa* rhizome has traditionally been used to reduce dysmenorrhea, as an analgesic, antipyretic and anti-inflammatory, as well as to treat colds, coughs, asthma, gastrointestinal and uterine diseases [2]. In spite of the potential anticancer activity from the rhizome, there is very little known about molecular information regarding the genetic character of *C. aeruginosa.* However, genetic information such as transcriptome data is not yet available. Therefore, these data were used to obtain transcriptome information from rhizome of *C. aeruginosa*. The transcripts results were obtained using short-read sequencing from the Illumina NextSeq 550 sequencer with PE150 mode. This data is able to provide transcripts that are useful for studying gene expression analysis and also for EST-SSR development.

## 2 Data Description

Here RNA-seq reads for rhizomes harvested from 6-month-*old C. aeruginosa* plants. To guarantee data reliability, quality control (QC) is performed at every step of the procedure from RNA sampling to final data, every step of the way, including sample testing, library preparation, and sequencing. The raw data obtained from the Illumina NextSeq 550 with PE150 platform were deposited as a FASTQ format in NCBI's Sequence Read Archive (SRA) repository under BioProject accession number PRJNA918644. Sequencing data analysis raw and clean reads were performed as shown in Table 1. The quality of the net reads was assessed, and a high-quality percentage of net reads were obtained. The high-quality reads were assembled to generate the contigs. De novo assembled was performed using rnaSPAdes 3.15.3 on Galaxy platform (toolshed.g2.bx.psu.edu/repos/iuc/rnaspades/rnaspades/3.15.3+galaxy2). The statistics of contigs assembling and contigs mapping was estimated (Table 2). The BUSCOs searches of complete (82.7%), fragmented (3.5%), and missing single-copy orthologs (13.8%) revealed a high level of gene completeness, supporting the excellent quality of the transcriptome (Table 3).

**Table 1:** Statistics of sequencing reads.

Parameters	Statistics
Total Raw Reads	42831232
Total Clean Reads	42053314
Total Raw Bases (Gb)	12.8
Total Clean Bases (Gb)	12.6
Clean Reads Q20 (%)	98.13
Clean Reads Q30 (%)	94.62
Clean Reads Ratio (%)	98.18
Error (%)	0.02
GC(%)	49.78

**Table 2:** Statistics of contigs.

Parameters	Statistics
Contig L50	30956
Contig N50	1661
Contig L90	110677
Contig N90	349
Contig len_max	15599
Contig len_min	74
Contig len_mean	964
Contig len_median	535
Contig len_std	983
Contig num_bp	164256987
Contig num_seq	170433
GC (%)	44.32

**Table 3:** Statistic of BUSCOs.

Parameter	Statistic	
Complete BUSCOs	1925	82.70%
Complete and single-copy BUSCOs	759	32.60%
Complete and duplicated BUSCOs	1166	50.10%
Fragmented BUSCOs	82	3.50%
Missing BUSCOs	319	13.80%
Total BUSCO groups searched	2326	

## 3 Experimental Design, Materials and Methods

### 3.1 Sample Sites Taken

**Fig. 1:**
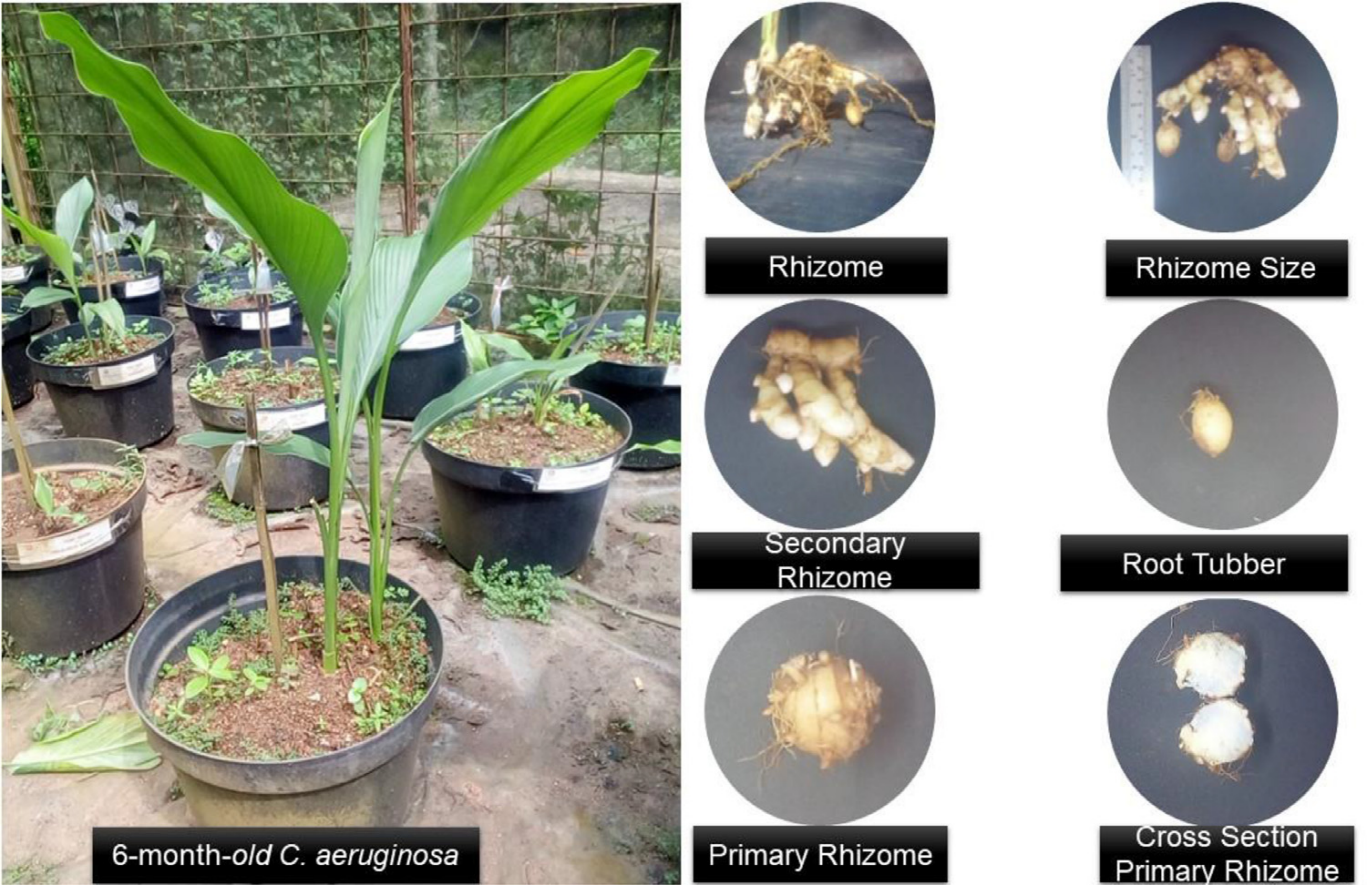
Phenotypic images of the medical plant *Curcuma aeruginosa* Roxb used in this study.

*C. aeruginosa* is grown in the greenhouse at Balai Bioteknologi-BRIN in South Tangerang, West Java, Indonesia, in pots filled with medium combination with ratio manure: soil: husk charcoal (2: 1: 1) (Fig. 1). Maintenance and fertilization are carried out based on medicinal plant cultivation standards.

### 3.2 RNA Extraction and cDNA Synthesis

The rhizomes were harvested from 6 months old plants and were immediately stored at −80 °C until RNA extraction. Following the technique, the total RNA was extracted using RibospinTM Plant-GeneAll. RNA degradation and contamination was monitored on 1% agarose gels. RNA purity was checked using the NanoPhotometer^Ⓡ^ spectrophotometer (IMPLEN, CA, USA). RNA integrity and quantitation were assessed using the RNA Nano 6000 Assay Kit of the Bioanalyzer 2100 system (Agilent Technologies, CA, USA).

The mRNA library was purified using the TruSeq RNA sample preparation v2 kit (Illumina Inc, CA, USA) according to the manufacturer's protocol. The library was checked with Qubit and real-time PCR for quantification and bioanalyzer for size distribution detection. Quantified libraries were pooled and sequenced on Illumina platforms, according to effective library concentration and data amount. The clustering of the index-coded samples was performed on a cBot Cluster Generation System using PE Cluster Kit cBot-HS (Illumina) according to the manufacturer's instructions. After cluster generation, the library preparations were sequenced on an Illumina platform (Illumina NextSeq 550 with PE150 platform) and paired-end reads were generated.

### 3.3 Data Analysis

Raw paired-end sequences were uploaded as FASTQ files to the Galaxy bioinformatic platform version 23.0.rc1 (https://usegalaxy.org/). Raw data (raw reads) of FASTQ format were firstly processed used FASTQC v0.73 to do quality control [3]. Clean data (clean reads) were obtained by triming reads containing adapter and removing poly-N sequences and reads with low quality from raw data. The trimming was done using Trimmomatic v0.38 [4]. At the same time, Q20, Q30 and GC content of the clean data were calculated using Fasta Statistics Galaxy v2.0. All the downstream analyses were based on the clean data with high quality a minimum length of 200 bp. The transcriptome was assembled using rnaSPAdes 3.15.3 in Galaxy at type of paired end: default (–pe); orientation of reads: FR (-><-); an additional set of short-reads: disabled; k-mer detection option: auto; Phred quality offset: auto; strand specificity: disabled and a minimum length of 150 bp using high-quality reads [5]. The *C. aeruginosa* transcriptome assembly was examined with BUSCO analysis at the conclusion of this procedure.

## CRediT authorship contribution statement

**Devit Purwoko:** Conceptualization, Methodology, Writing – original draft, Funding acquisition. **Imam Civi Cartealy:** Supervision. **Siti Zulaeha:** Data curation, Investigation. **Teuku Tajuddin:** Project administration, Writing – review & editing. **Gemilang Rahmadara:** Writing – review & editing. **Nurul Fitri Hanifah:** Data curation. **Endah Dwi Hartuti:** Data curation. **Hayat Khairiyah:** Data curation. **Amila Pramisandi:** Supervision. **Yusuf Sigit Ahmad Fauzan:** Resources. **:** Resources. **Rikania Reninta:** Validation. **Anna Safarrida:** Investigation. **:** Supervision.

## Declaration of Competing Interest

The authors declare that they have no known competing financial interests or personal relationships that could have appeared to influence the work reported in this paper.

## Data Availability

RNA dataset from C. aeruginosa (Original data) (https://www.ncbi.nlm.nih.gov/sra/PRJNA918644).
